# Designing practical science assessments in England: students’ engagement and perceptions

**DOI:** 10.1080/02635143.2021.1872519

**Published:** 2021-01-22

**Authors:** Yasmine H. El Masri, Sibel Erduran, Olga Ioannidou

**Affiliations:** a Department of Education, University of Oxford, Oxford, UK; b The Norwegian Centre for Science Education, University of Oslo, Oslo, Norway

**Keywords:** Practical science, summative assessment, diversity of scientific methods; GCSE

## Abstract

**Background:**

The paper discusses how the design of summative assessments of practical science can be enhanced through the use of robust theoretical models that capture the diversity of scientific methods. The developments in assessment policy in England and the role of practical science in teaching and learning of science are reviewed.

**Purpose:**

The paper has two main purposes: (a) to explore how summative assessments of practical science can potentially reflect the breadth of scientific methods; and (b) to investigate how Year 10 students in England engage with and perceive summative assessments designed using Brandon’s Matrix, a framework clarifying the diversity of scientific methods.

**Sample:**

The paper draws on data from Year 10 (14–15 years old) students with a sample of physics assessment tasks developed using Brandon’s Matrix.

**Design and methods:**

The study focused on the development of a series of assessment questions based on Brandon’s Matrix, a framework by a philosopher of science, who classifies scientific methods in a taxonomy. Students’ performance on the assessments as well as their perceptions about practical science are investigated.

**Conclusion:**

The results suggest that students held a very narrow understanding of what a scientific method is and often linked it to hypothesis testing, variable manipulation and measurement of several variables. Furthermore, they reported not to enjoy drawing conclusions from data.

## Introduction

In the current climate of science education reform, the assessment of practical science has become a key concern. In the USA, the publication of ‘A Framework for K-12 Science Education: Practices, Crosscutting Concepts and Core Ideas’ (National Research Council 2012), culminated in the new science education standards represented by the *Next Generation Science Standards* (NGSS Lead States 2013). Pellegrino et al. (2014) believe that four aspects of the framework’s vision for science education require significant change in approaches to assessment:

“(a) a focus on developing students’ understanding of a limited set of core ideas in the disciplines and a set of crosscutting concepts that connect them;

(b) an emphasis on how these core ideas develop over time as students’ progress through the K-12 system and how students make connections among ideas from different disciplines;

(c) a definition of learning as engagement in the science and engineering practices, to develop, investigate, and use scientific knowledge, and

(d) an assertion that the science and engineering learning for all students will entail providing the requisite resources and more inclusive and motivating approaches to instruction and assessment, with specific attention to the needs of disadvantaged students.” (p. 26)

Such recommendations can be integrated into educational assessments which ultimately determine what is valuable to learn by allocating credit to selected aspects of what students know and can do. However, the design of assessments, especially high-stakes summative assessments, is not always defined in terms of what best promotes students’ learning. Indeed, the relationship between the design of assessments and a learning theory is hard to establish (James 2008). Baird et al. (2017) outlined some of the past attempts to associate learning theories with assessment design. For instance, behaviourist learning theories (e.g., Bandura 1969; Skinner 1953; Watson 1930) which focus on promoting desired behaviours and discouraging undesired ones with less interest in thought processes have been linked to controlled assessments and multiple-choice tests where only performance mattered (James 2008). In contrast, cognitive learning theories (e.g., Neisser 1967; Sternberg 1981), which underlie some current assessment systems (Haladyna and Rodriguez 2013), give precedence to the psychological processes involved in learning. The assessments emphasise meaning making and conceptual understanding with partial credit being allocated to elements of the response such as the use of a correct approach for answering a question (James 2008). Lastly, socio-constructivist theories of learning underscore the importance of the learner’s interactions with the surrounding environment and with others in building knowledge and skills (Vygotsky 1978). These theories underlie assessment formats such as portfolios, peer assessment and reflective diaries (e.g., Black and Wiliam 1998b, 1998a; Shepard 2000; Torrance and Pryor 1998).

While efforts have been made to relate assessments to certain learning theories, these relationships have been inferred *post hoc* and not at the outset. i.e., at the design and development stage of the assessment. The challenge of prioritising learning theories in the design of assessments becomes even more prominent when the focus of learning is on complex thinking. The assessment of complex thinking has been impeded by various factors including the prohibitive cost and time needed to mark such assessments as well as the accountability pressures which often narrow the curriculum to can be easily and manageably assessed (Ercikan 2015). In England for instance, such pressures have led to reforms that changed the nature and format of practical science high-stakes assessments at age 16 (Ofqual 2013, 2015).

In this paper, we extend previous efforts in designing assessments of complex thinking in science (Pearson et al. 2015; Ryoo and Linn 2015) by proposing a new framework for assessing practical science that relies on a robust theoretical model that aims to clarify what is meant by scientific methods. The paper utilises a theoretical framework derived from Brandon’s Matrix (Brandon 1994) which clarifies the diversity of methods used in science. The theoretical model, which will be described in the rest of the paper, is robust in the sense that it has been well argued in philosophy of science (Brandon 1994) and also applied in framing theoretical (Erduran and Dagher 2014) as well as empirical (Cullinane, Erduran, and Wooding 2019) studies in science education. Considering the vast variation in science assessment systems around the world, we focus our discussion on the assessment landscape of one country so as to illustrate in-depth the existing challenges such as reliability and validity (e.g. Ofqual 2013) and how these challenges can be addressed in terms of design of summative assessments. The paper has two main purposes: (a) to explore how summative assessments of practical science can potentially reflect the breadth of scientific methods employed by scientists; and (b) to investigate how Year 10 students in England engage with and perceive summative assessments that incorporate such breadths of scientific methods as exemplified by Brandon’s Matrix.

The paper begins by examining assessment policy reforms in the context of England. We argue that the theory underlying the learning of practical science is incongruent with the latest approaches to design of the assessments. We then propose a framework for designing assessments that are in line with the current curriculum reform policy and grounded in robust theoretical frameworks. The paper goes on to address the first research question and describes the development of a series of assessment questions based on a framework of scientific methods developed by a philosopher of science, Brandon (1994). The overall approach aims to expose students to the plethora of scientific methods adopted in science inquiry and to engage their thinking skills making the summative assessment a *test worth teaching to* (Linn 2000; Popham 1987; Shepard 2000). The empirical component of the paper addresses the second research question and provides insight into the performance and engagement of Year 10 (14–15 years old) students with a sample of physics assessment tasks developed by using Brandon’s framework.

## Literature review

### High-stakes summative assessments in England

Curriculum assessments target the knowledge and skills that are described in the curriculum and that students are expected to have learnt in school. In England, the focus and content of the curriculum and its assessment are typically defined at policy level. The design of Key Stage 4 science curriculum and assessment has evolved over time and new policies have been introduced in 1992, 2006 and 2016 since the inception of the General Certificate of Secondary Education (GCSE). GCSEs refer to academic qualifications that 16-year-olds in England, Wales and Northern Ireland gain upon passing high-stakes national examinations in subjects of their choice.

In a recent article, Childs and Baird (2020) presented a historical review of assessment policies in England and identified three periods: the first being from 1992 to 2006, the second from 2006 to 2016 and the third from 2016 until present. During the first policy period (1992–2006), the assessment of practical work consisted of science investigations assessed by teachers throughout the course; in the classroom or as homework. The design of this assessment mirrored the curriculum developers’ intentions to promote creative and innovative ways of teaching and assessing science that reflected how scientists work (Donnelly et al. 1996; Jenkins 1995).

The emphasis on the construction of knowledge and the negotiation of meaning with peers in the assessment suggests that elements of socio-constructivist learning inspired its design. Students were supposed to be assessed based on what they learned by *doing* science and interacting with their peers. However, as Childs and Baird (2020) detailed in their document analysis, many contextual factors including teachers’ lack of expertise and training in assessing science investigations as well as the high assessment burden that this design entailed, led to the policy being implemented in an overly prescriptive way in schools. Instead of creating an environment that encouraged thinking and building understanding by negotiating meaning with peers, science investigations were reduced to students filling writing frames and ticking boxes (Jenkins 1995). This has resulted in the opposite of the intended effect where students’ experience of practical work became narrower leading to concerns over the validity of the assessment.

The 2006 policy aimed to address the unintended negative effects of the 1992 policy by providing more space for the nature of science advocated in Millar and Osborne’s (1998) *Beyond 2000* report and by introducing regulations to limit malpractice and grade inflation. During the second policy period (i.e., 2006–2016), the assessment of practical science was conducted by teachers as controlled assessments in the classroom under supervision. The tasks varied across examination boards with a combination of practically based and non-practically based tasks which included complete investigations, case studies, data analysis, research tasks, portfolios, etc. While these assessments were not necessarily designed to be carried out in groups and foster socially situated learning, they were still inspired by constructivist ideas that valued students’ experiences and learning by doing.

Nevertheless, as Childs and Baird (2020) explained, the new regulations put in place in the 2006 policy were unsuccessful in addressing the issues that had emerged in the first policy period. Teachers’ workload and low level of expertise, the narrowing of the science curriculum as well as the validity and reliability issues remained the centre of much concern (Ofqual 2013, 2015). This has led to the introduction of a new policy in 2016 that embedded the assessment of practical science within the GCSE science papers in the form of written examinations. In the new assessments, students needed to demonstrate their understanding of scientific enquiry and questions probed their experiences of practical work (Ofqual 2015).

The new approach to the assessment of practical science, like all GCSE papers, awards marks to cognitive processes and methods rather than merely giving credit to correct responses. This suggests that the assessment implicitly rests on cognitive learning theories. However, Childs and Baird (2020) point to some teaching guidance provided by examination board that may unintentionally encourage a teaching approach of practical work that has been long criticised for being formulaic. In this approach, students conduct practical work by mindlessly following instructions as if they were steps of a cookbook recipe until they reach an outcome that has been often predicted for them (Clarkson and Wright 1992). The focus on students’ *correct* behaviour and outcome aligns this approach well with behaviourist learning theories.

With the assessment of practical science moving away from the socio-constructivist theories that motivated its progressive design and implementation in the early 90s due to the practical considerations associated with the negative impact of high-stakes examinations, it is important to ensure that the current design of the assessment still encourages deep learning. Designing an assessment includes decisions around what to assess (i.e., the construct) and how best to assess it. Wiliam (2010) argued that the construct should be the priority as the way it is operationalised in the assessments determines the knowledge and skills that students have to acquire to pass the test and hence impacts on the learning and teaching. This impact, commonly referred to as *washback* or *backwash* (Alderson and Wall 1993), can be unintended and negative especially in high-stakes contexts where the taught curriculum is narrowed to what is assessed (Au 2007; Madaus, Russel, and Higgins 2009; Stobart and Eggen 2012).

### Teaching and learning of practical science

In this section, we focus more closely on characterisation of practical science. In England, the term ‘practical science’ has been widely used in characterizing aspects of scientific methods. The term ‘practical science’ as ‘ … a shorthand for the full programme of experimental and investigative activities (including fieldwork) conducted as part of science education in schools and colleges’ (House of Lords Science and Technology Committee 2006, 63). In the past years, practical science has been considered as a key part of the science curriculum and has been promoted as an authentic demonstration of scientific phenomena and practices allowing students to engage in empirical enquiry (Osborne 2015). In a comprehensive literature review, Hofstein and Lunetta (2004) identified five main educational goals for teaching practical science: a) understanding of scientific concepts; b) interest and motivation; c) scientific practical skills and problem-solving abilities; d) scientific habits of mind and e) understanding of the nature of science. The goals are reflected to a large extent in England’s Department for Education (2015) ‘Working Scientifically’ (WS) learning and assessment objectives which include: a) development of scientific thinking; b) experimental skills and strategies; c) analysis and evaluation; and d) scientific vocabulary, quantities, units, symbols and nomenclature. This suggests that the emphasis of practical science curricula and assessments in England is not limited to practical manipulation skills (e.g. use of equipment) but also includes the promotion of students’ higher order thinking skills, such as problem-solving and scientific thinking.

Nevertheless, as described in the previous section, the accountability pressures resulting from the inclusion of practical work in GCSE science assessments distorted the role that practical work played in science instruction and impacted on the nature of the practical investigations conducted in classrooms, making them overly prescribed and oriented towards ensuring that students performed well in the assessments (Childs and Baird 2020). This has limited teachers’ creativity in using practical science in the classroom as a way to engage students in learning how science really works, as well as to motivate their curiosity and enhance their inquisitiveness (Abrahams, Reiss, and Sharpe 2013).

In the same vein, researchers have raised questions about the extent to which practical work, typically focused on *a hands-on* approach to science, promoted the learning and teaching of science (Abrahams 2009; Abrahams and Millar 2008). Criticism included the *cookbook* approach to teaching practical work where students followed a set of instructions mindlessly, without understanding the rationale behind the steps taken and the purpose of the practical (Erduran and Dagher 2014). Relevant studies have shown that this routinised way of doing science resulted in students’ difficulties in justifying their claims, as well as in seeking for explanations (Watson, Swain, and McRobbie 2004), while students’ discussions were limited in practicalities about how to carry out a given investigation (Abrahams and Millar 2008).

Another criticism of the value of practical work in learning science related to the overemphasis of a single valid method for scientific inquiry, often referred to as *the scientific method* which vastly uses experimentation or hypothesis testing methods. This method inaccurately portrays a linear process of science inquiry which often begins with the formulation of a hypothesis and ends with the communication of a conclusion that confirms or refutes the hypothesis (McComas 1998; Wivagg and Allchin 2002). This makes the learning process monotonous and often predictable; and hence strips learning science from its inquisitive nature (Erduran and Dagher 2014). In response to such a formulaic approach, Abrahams (2010) had argued for a more *minds-on* approach to learning science, which would offer learners the opportunity to be exposed to and reflect on a range of aspects of science, including the methods used when *doing* science and hence to develop epistemic, as well as procedural understanding of science.

Given the interplay between teaching and assessment, one would expect that the idea of a single, linear scientific method would be mirrored in design of high-stakes assessments for practical science. If tests are designed in favour of specific scientific methods, such as experimentation and hypothesis testing, teachers are more likely to favour these methods in their teaching. In a recent study, Cullinane, Erduran, and Wooding (2019) reported an imbalance in the representation of scientific methods in chemistry GCSE papers in England, highlighting that although classroom teaching tends to emphasise hypothesis testing and manipulation of variables as key aspects of the scientific method, the assessments mainly focused on manipulative parameter measurement. The findings also indicated that more marks were dedicated to hypothesis testing and manipulation of variables, raising questions about the assumptions being made about different scientific methods and the corresponding cognitive demands. The authors called for a more balanced representation of the methods commonly used in science in the design of high-stakes summative assessments.

### Designing summative assessments of practical science

The new educational policy of the GCSE in England resulted in many reforms in the science curriculum and the design of the assessments, including the assessment of practical science (Ofqual 2015). The 2016 assessment reform was informed by Ofqual’s wide consultations with a range of stakeholders including examination boards, awarding bodies, teachers, schools, employers, local authorities, educational specialists and was a response to multiple issues identified in the pre-existing assessment regime. In the decade preceding the reform, practical science assessment consisted of controlled assessment. i.e., coursework conducted in the classroom under teachers’ supervision. Despite the quality control procedures put in place by examination boards, including the external moderation of marking, this approach resulted in a number of challenges such as grade inflation (Coe 2007) and cheating which undermined the credibility of the assessment and serious concern was raised over its validity and reliability (Ofqual 2013). The assessment was not assessing what it was supposed to assess thereby compromising its validity and it was not delivered and marked consistently thereby lowering its reliability. Moreover, its organization was laborious and time-consuming, reducing the time that could be more effectively used for teaching and learning. The new model of assessment introduced in 2016 ensured that the aims of the science curriculum were delivered and that students were provided with opportunities of engaging with a wide variety of practical work, while making sure the assessment was manageable for schools, valid and reliable (Ofqual 2015). As a result, the assessment of science practical work was embedded within the written GCSE science papers accounting for 15% of the total marks available and assessed students’ understanding of the science practicals they carried out in class rather than their manipulative skills in carrying out practical work.

At this stage, it is useful to refer to the distinctions about practical science provided by Reiss, Abrahams, and Sharpe (2012). These authors make a useful distinction can be made between direct assessment of practical skills (DAPS) and indirect assessment of skills (IAPS). The former, DAPS, refers to any form of assessment that requires students, through the manipulation of real objects, to directly demonstrate a specific or generic skill in a manner that can be used to determine their level of competence in that skill. An example of this would be if a student was assessed on their skill in using an ammeter and this was determined by requiring them to manipulate a real ammeter and use it within a circuit to take readings and for these readings need to be within an acceptable range of values for the student to be credited. In contrast, IAPS relates to any form of assessment in which a student’s level of competency, again in terms of a specific or generic skill, is inferred from their data and/or reports of the practical work that they undertook. For example, when a student writes up an account of the reaction between hydrochloric acid and calcium carbonate chips in a way that the marker would not be certain if the student is writing what they have just done or simply remembering what they have previously done or been told about this reaction (Reiss, Abrahams, and Sharpe 2012).

In order to ensure that written GCSE assessments of practical science are designed in a way to reflect a balance in the representation of scientific methods and strengthen construct validity as advocated by Cullinane, Erduran, and Wooding (2019) and differentiated by Reiss, Abrahams, and Sharpe (2012), we adopt Erduran and Dagher (2014) proposal of using Brandon’s (1994) Matrix as a framework in order to classify experimental and non-experimental scientific methods in a two-by-two matrix (Table 1). Brandon’s Matrix provides a summary of scientific methods by a taxonomy about the diversity in scientific investigations (Brandon 1994). According to Brandon, scientific investigations can be classified according to two criteria: (a) whether they are experimental (i.e., whether or not a variable is manipulated) and (b) their aims (whether it includes hypothesis testing or parameter measurement).

**Table 1.** Brandon’s matrix (reproduced from Brandon 1994, 63).

	Manipulate	Not manipulate
Test hypothesis	Manipulative hypothesis testing	Non-manipulative hypothesis testing
Measure parameter	Manipulative description or parameter measurement	Non-manipulative description or parameter measurement

**Figure 1.**
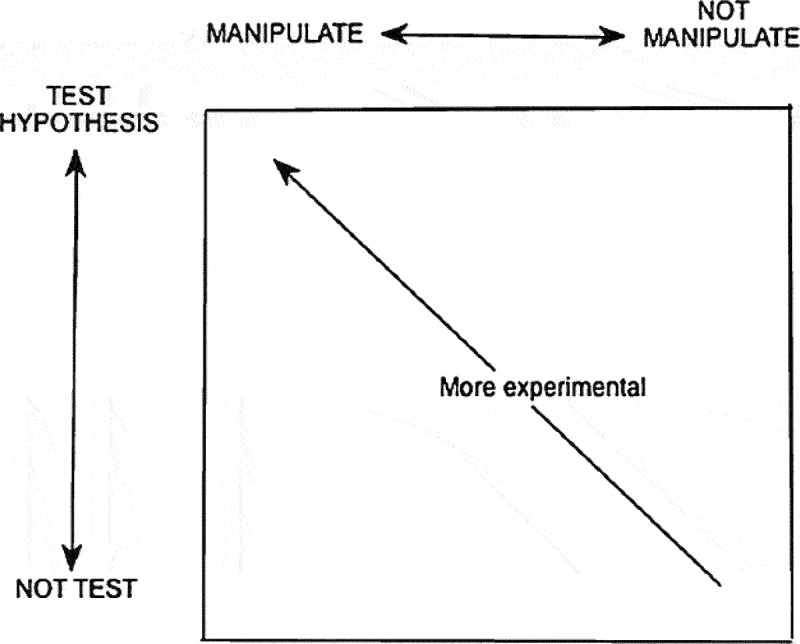
Brandon’s representation of the ‘space of experimentality’ between two continua (Brandon 1994, 66).

Brandon (1994) noted that scientists use a variety of scientific methods to draw conclusions and answer their questions and their preference of one method in a particular study is dictated by their research questions, resources and expertise rather than a hierarchy making some methods more reliable than other in absolute terms. In addition, Brandon highlighted that the lines separating the different categories in the matrix are not rigid and that one has to imagine scientific methods as existing in a ‘space of experimentality’ (66) defined by two continua as shown in Figure 1 (*not manipulate – manipulate* and *not test hypothesis – test hypothesis*).

In the rest of this paper, we describe the design of science assessments based on Brandon’s Matrix and present an empirical study conducted with Year 10 students in England to investigate how students engaged and perceived these assessments.

## Methodology

### Research questions

The empirical study was guided by the following research questions:

1. How can the design of summative assessments of practical science reflect the breadth of scientific methods employed by scientists?
2. How do Year 10 students in England engage with and perceive summative assessments designed using Brandon’s matrix that illustrates the breadth of scientific methods?

### Designing assessments

The research was conducted in the context of Project Calibrate based at University of Oxford (Erduran, 2020). Six science assessments were developed with their mark schemes by professional examiners who are experts in writing science questions for GCSE assessments in England. The assessments were developed in a way to be aligned with Key Stage 4 (age 14–16) science curriculum and the assessment objectives outlined in Table 2 (DfE 2017).

**Table 2.** Assessment objectives for Key Stage 4 in England.

AO1	Demonstrate knowledge and understanding of scientific ideas and scientific techniques and procedures
AO2	Apply knowledge and understanding of scientific ideas scientific enquiry, techniques and procedures
AO3	Analyse information and ideas to interpret and evaluate, make judgements and draw conclusions, develop and improve experimental procedures.

In addition to being aligned with Key Stage 4 science curriculum and assessment objectives, the assessments were designed using Brandon’s (1994) Matrix as a guiding framework. Prior to designing the assessments, examiners attended three workshops, each one a day-long to introduce them to Brandon’s Matrix (Wooding, Cullinane, and Erduran 2020) and support them with the development of questions using the framework. Each assessment consisted of a set of five questions mapped on Brandon’s matrix and targeting one of six topics of practical science: *Plant growth and distribution* and *Osmosis* in biology, *Chromatography* and *Mixtures and distillation* in chemistry and *Electrical circuits* and *Electromagnetic spectrum* in physics. The assessments can be accessed at the following website: ProjectCalibrate.web.ox.ac.uk. These topics selected are included in the national science curriculum for England (DfE, 2015, 2017).

The first four questions targeted a specific quadrant of Brandon’s matrix while the fifth question targeted more than one quadrant and required students to compare different scientific methods. In other words, the fifth question was a higher level question requiring understanding of all of Brandon Matrix categories. It required students to compare and contrast the different scientific methods and thus engage in higher order thinking strategies such as synthesis and analysis. For the purpose of this paper, we will only focus on how students engaged with the first four questions of a physics assessment assessing the topic of electrical circuits. Teachers were recruited to administer the questions in their lessons in a mock examination context. The questions were selected by the participating teachers based on their teaching schedule at the time of the study. Each question included three to six sub-questions in each. Question 1 was a manipulative hypothesis testing task where students examined the relationship between the variation of the length of a piece of wire and its resistance. Question 2 was a non-manipulative parameter measurement question where students were expected to determine the resistance of a piece of copper wire based on a plot of the current as a function of the potential difference. Question 3 was a non-manipulative hypothesis testing question where students were asked to provide evidence in support of a hypothesis explaining the variation of the current in a filament lamp over time. Question 4 was a manipulative parameter measurement question where students are asked to explain the relationship between bone growth, the size of the current passing through the body and the reading of an electronic scale. The maximum score was 31 marks evenly distributed across the four questions with 7 or 8 marks available for each question. All sub-questions required constructed responses, some being as short as a numerical figure while others being more extended paragraphs. Once developed, the assessments went through a series of revisions and refinement involving examiners and researchers to evaluate the quality of the questions.

### Data collection

Although the assessments were intended for Year 11 (15–16 year old) students, the challenge of recruiting students in their examination year meant that we administered the assessments to a sample of Year 10 students and subsequently gathered their perceptions of the assessment using surveys. In order to minimize impact on the validity and reliability of the assessments, we ensured with the help of teachers that students completed assessments on topics they had already learned. Although the age group not being those who typically take the high-stakes GCSE examinations is alimitation of the study, we believe that the findings of this research with slightly younger students can still be informative in refining assessments and gaining understanding of how students engage with such assessments. In the next section, we describe the research instruments and methods used to collect and analyse the data.

#### Students’ performance on the assessments

The physics assessment was administered in two state secondary schools in England with a total of 52 Year 10 students, 24 in one school (S_1_) and 28 in the other (S_2_). Two of the participating science teachers introduced Brandon’s (1994) Matrix within the context of the lesson on electrical circuits and later administered the assessment. Teachers were advised to administer the assessments under examination conditions (i.e., students solve all the questions individually, without consulting any textbook or notes and within a specific duration).

Using the mark scheme, two markers who are former physics teachers and experts in science education marked the students’ scripts independently. The inter-rater reliability was high with a Pearson correlation R = 0.93 suggesting that the mark scheme worked well and that there is high consistency in the marking between the two markers. Discrepancies in the total scores were reconciliated by computing the average of the two total scores if the discrepancy was within three marks. Otherwise, students’ scripts were remarked, and new final scores were generated.

#### Students’ perceptions of the assessments

Following the administration of the physics assessment, Year 10 students in both participating schools were queried in order to explore their perceptions of the assessments using a survey that was developed by the project researchers and consisted of three main sections: (1) students’ attitudes towards practical science; (2) students’ perceptions of the science assessments; and (3) students’ understanding of Brandon’s Matrix. The surveys included three types of questions: 5-point Likert scale questions, multiple choice questions and short constructed response questions. Table 3 provides examples of the survey questions administered to students after sitting the assessment.

**Table 3.** Examples of survey questions administered to students.

Survey section	Examples of survey questions
Attitudes towards practical science	How much do you agree or disagree with the statements below? Please select one response in each row [Strongly disagree … Strongly agree].
Perceptions of the science assessments	What did you find most challenging in the test questions?
Understanding of Brandon’s matrix	In which scenario did the student test a hypothesis? □ Scenario 1□ Scenario 2□ Both□ Neither Please give at least one reason for your answer.

### Data analysis

Assessments of high quality include easy and more difficult questions to offer students of different proficiency levels the opportunity to demonstrate their learning of the assessed topic and to allow the discrimination between those who have achieved the targeted standard from those who exceeded it. In order to assess the extent to which the assessment developed includes questions of a range of difficulty, we examined the distribution of students’ total scores and computed descriptive statistics such as the mean, standard deviation and completion rates. For each of the survey questions, the frequency of responses was tallied, and proportions of responses were computed and sometimes presented for each gender.

## Results and findings

### Difficulty of the assessments

**Figure 2.**
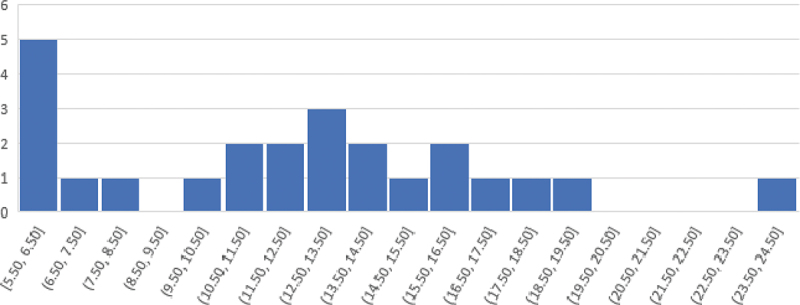
Distribution of total scores.

Assessments are often developed in a way where total scores are normally distributed; that is, in a way where most test-takers are expected to score around the mean score (i.e., average) with fewer students scoring below and above the mean. In this study, most students (18 students) scored below 50% of the 31 marks available; and less than a third of the students (6 students) scored above 50% (Figure 2).

These descriptive statistics in Table 4 suggest that the assessment was hard for the participating students. The mean score was 12.3; 3.2 points lower than the expected mean score of 15.5 representing 50% of the marks. Similarly, the median score was 12.5 indicating that 50% of the participating students scored below 12.5 marks. The standard deviation from the mean score was 4.9; this is a large value indicating a wide spread of scores (~5 marks) around the mean. Likewise, the high dispersion of scores can be also observed in the wide range of 19 marks between the lowest score of 5.5 (18%) and the highest one of 24.5 (79%) suggesting that the performance was very heterogeneous within the group.

**Table 4.** Descriptive statistics.

Statistic	Value
Mean	12.3
Standard deviation	4.9
Median	12.5
Minimum score	5.5
Maximum score	24.5
Range	19.0

In addition to the descriptive statistics provided in Table 4, we examined the completion rates for every question, that is, we computed the percentage of students who completed each question by subtracting the percentage of missing responses for every question from 100. Completion rates are expected to be above 90% for easy to average questions. Out of a total of 23 questions, 9 questions were completed by less than 90% of students, five of which were completed by less than 80% of students. The location of these items ranged between being at the end of a particular task or at the end of the assessment. Lower completion rates are a sign of ambiguity or difficulty in a question or the effect of speediness if the question is located at the end of the test (Borghans and Schils 2019).

The results are not entirely surprising given that the students had very limited exposure to teaching based on Brandon’s matrix and participating students were in Year 10 while the assessment questions were aimed at students at the end of Year 11. Teaching time, age group and familiarity with the type of questions are likely to have affected the demands and ultimately the relative difficulty of the questions as reported in other studies (Kyriakides and Creemers 2009; Muijs and Reynolds 2010; Reeve, Heggestad, and Lievens 2009). Had the students been exposed to teaching approaches based on Brandon’s Matrix, with repeated reinforcement across the school year leading up to the examinations, we would have expected that the factor of unfamiliarity would have been eliminated and the students were more likely to find the questions easier to respond to.

### Students’ perceptions of the assessments

Forty-eight students from two schools (n_1_ = 22 and n_2_ = 26) took part in the survey. The sample was gender-balanced with 20 males and 22 females taking part with six participants not indicating their gender.

#### Interest in practical science

**Figure 3.**
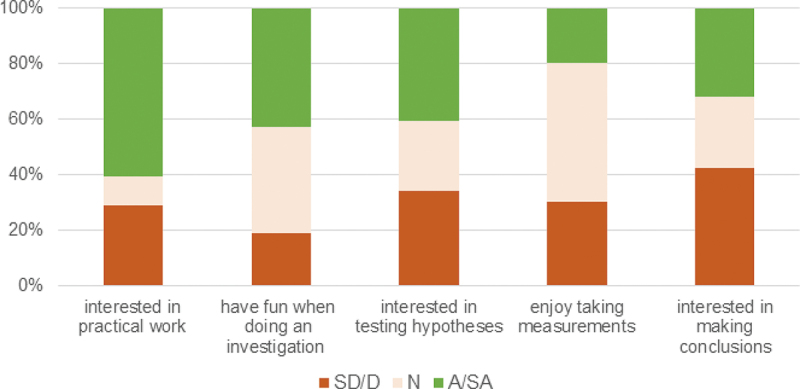
Students’ attitudes towards science practical (n = 48).

Based on Figure 3, out of 48 students, 29 (60%) agreed or strongly agreed with the first statement *‘In general, I am interested in doing practical work’* while 14 (29%) disagreed or strongly disagreed with this statement. In addition, 20 students (42%) agreed or strongly agreed with the statement *‘I generally have fun when I am doing an investigation’* while 9 (19%) disagreed or strongly disagreed with this statement. Interest in a subject and the enjoyment experienced underlie self-determinate and intrinsic forms of motivation to learn the subject (Krapp and Prenzel 2011). Intrinsic motivation affects the extent of student engagement and performance (Ryan and Deci 2000). Schiefele, Krapp, and Winteler (1992) meta-analysis suggests that, for all subjects and year levels, interest in a subject is correlated with academic performance (R = 0.30) with this correlation being amongst the highest in science and in particular physics. The data suggests that nearly a third of the students who completed the survey expressed a low level of interest in doing practical work with 19% expressing a lack of enjoyment in carrying out investigations and over a third conveying a level of indifference towards carrying out investigations. Our results are consistent with those observed by Sharpe and Abrahams (2020). These authors reported that secondary students’ attitudes to practical work were positive they were not constant and homogenous but change over time. The affective value of practical work was found to vary by subject although in all three sciences this value decreased as students approached their GCSE examinations.

SD = Strongly disagree; D = disagree; N = neutral; A = Agree; SA = Strongly agree

Student responses to the statement *‘I am interested in testing hypotheses (predictions)*’ were equally split between students who disagreed or strongly disagreed with the statement (n = 16, 34%), those who agreed or strongly agreed with the statement (n = 19, 40%) and those who neither agreed nor disagreed (n = 12, 26%). Students are often exposed to hypothesis testing questions especially in contexts where *the* linear scientific method dominates teaching and curricula (McComas 1998; Wivagg and Allchin 2002). Based on this survey, such focus could alienate and disengage at least a third of students and hence have implications for their learning of the subject and their performance in it.

Half of the students (33 out of 46) indicated that they neither agreed nor disagreed with the statement *‘I enjoy taking measurements in a practical activity’*. Only nine students (20%) agreed or strongly agreed with the statement while the rest of the students (30%) disagreed or strongly disagreed with it. The high proportion of students expressing a neutral stance or a lack of enjoyment could be reflecting the less engaging cookbook recipe-like approach of teaching practical science (Clarkson and Wright 1992) where students are mechanically following instructions and filling out tables or plotting graphs without a deep understanding of the variable being measured or the procedure adopted to measure it.

A substantial proportion of students (20 out of 47, 43%) disagreed or strongly disagreed with the statement ‘*I am interested in making conclusions after a practical activity’* while 15 (32%) agreed or strongly agreed with it and 12 (26%) indicated a neutral position. Drawing conclusions is the most cognitively demanding activity amongst the three proposed in this question (testing hypotheses, taking measurements and making conclusions) because it relies on students making the correct observation or measurement and use their knowledge and thinking skills to arrive to a suitable conclusion.

**Figure 4.**
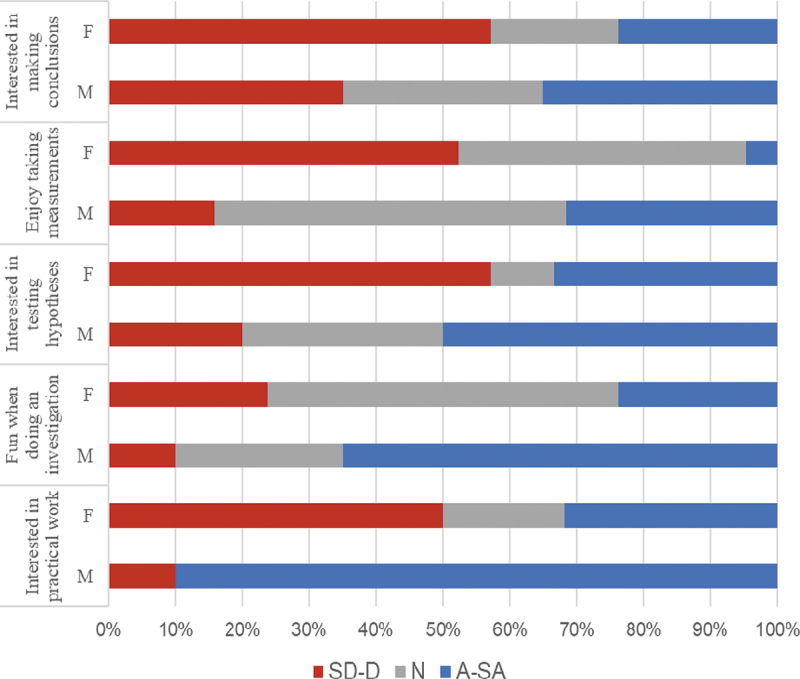
Gender differences in interest in practical science.

Gender differences were observed in responses to question 1 as presented in Figure 4. For all questions, frequencies of disagreement with the statements were higher for females than they were for males. Electrical circuit is a topic commonly favoured by males (Kang et al. 2019). While we acknowledge the small size of the sample, our findings seem to conform to previous research. Low levels of interest and enjoyment experienced by young girls in Science, Technology, Engineering and Mathematics (STEM) can deepen the already existing gender differences in performance in certain subjects and subsequent career choices. Sadler et al.’s (2012) study based on 6,000 students in the USA revealed large gender differences in STEM career aspirations during high school years, with males showing far more interest in engineering and females being attracted to medical and paramedical careers.

SD-D = Strongly disagree to disagree; A-SA = Agree to strongly agree; N = Neutral; M = Male; F = Female

#### Activity preferences

**Figure 5.**
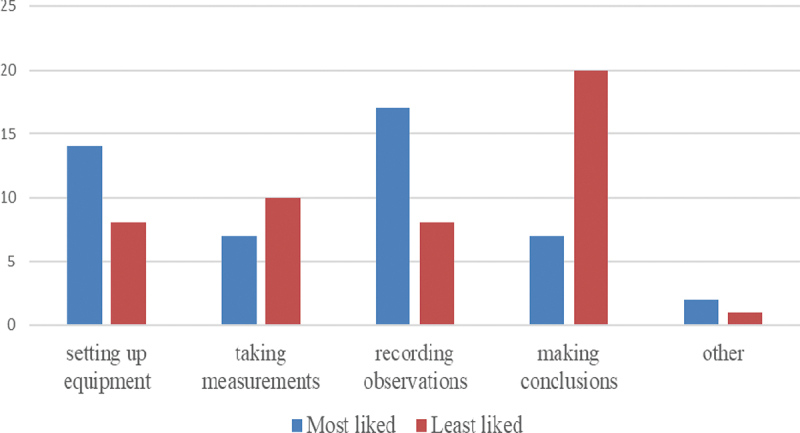
Most and least favoured practical activity.

Students were asked to indicate their favourite and least favoured practical science activity. Figure 5 presents the results based on 47 responses. Consistent with Question 1, taking measurements and making conclusions were amongst the least favoured activities in practical science. However, students reported enjoying setting up equipment and recording observations. These results reflect a contrast that is difficult to interpret without additional data. Taking measurements often entails manipulating equipment and recording observations so it is not clear what students were referring to when they selected their favourite and least favourite practical science activity.

#### Preparing for assessments

When students were asked to indicate what would have best prepared them for questions such as the ones they encountered as part of the study, the most common out of 46 responses was *‘doing similar questions in class’* (n = 20) followed by *‘doing more practical work’* (n = 10) and *‘revising what has been done in class’* (n = 9). Doing more of the same and repeating what has been done refer to drilling which is likely to be the test preparation strategy most students use when preparing for high-stakes assessments (Berliner 2011).

#### Opportunity to demonstrate understanding

When asked whether the assessments allowed them to demonstrate their understanding of practical science, only 14 out of 44 (32%) students said agreed while the other 30 (68%) disagreed citing reasons such as finding the questions poorly connected to exercises they carried out in class and difficulty in understanding the questions because of the ambiguity of the language. Indeed, when asked about the most challenging aspect in the assessments, most students (82%) reported that language as being a main impediment to understanding questions (e.g. ‘*The wording of the questions were a bit hard to understand but otherwise fine*’; ‘*Understanding what they are asking’*). In addition, two students reported finding the graphs complicated (e.g. ‘*Explaining what the data shows on a graph or calculations’*). Language and graphs have been shown to predict question difficulty in science assessment with them sometimes being the source of construct-irrelevant variance (El Masri et al. 2017; Ferrara and Duncan 2011). In this study, the development of the assessments was with experienced GCSE question writers. Moreover, a comparison of language complexity using CohMetrics (Graesser et al. 2014; Graesser and McNamara 2011) of the first two assessment tasks with two physics GCSE assessment tasks covering the same concepts suggests that the language complexity in the questions was consistently lower than that in GCSE assessments. The lack of understanding of questions is therefore most likely due to the novelty of the type of questions.

#### Understanding of Brandon’s matrix

In order to assess their understanding of Brandon’s Matrix, students were provided with two practical science scenarios followed by two questions. The first question required students to identify the scenario in which a student tested a hypothesis and changed one variable. The second question required students to reflect on whether the investigation described in the second scenario was *scientific* given that it did not test a hypothesis.

Most students (42 out of 46) were able to identify the scenario that tested a hypothesis and changed one variable and justify their choice (e.g., *She measured the plants at different intervals. Changing the variable*). However, in response to the second question, only five out of the 34 students who attempted the second question found that hypothesis testing was not necessary for an investigation to be considered scientific. They justified their answers by stating that the investigation involved recording (n = 2), measuring a variable (n = 2) or conducting an experiment (n = 1). The rest of the students (n = 29) thought that the investigation was not scientific and provided reasons summarised in Table 5. The results suggest that most students relate scientific methods with hypothesis testing (n = 6), measurement (n = 7) and manipulation of variables. A few students mentioned the lack of information about the experimental setup as their reason for judging James’s investigation as non-scientific.

**Table 5.** Summary of reasons provided by students.

Reasons for why second scenario is not scientific	Frequency
No hypothesis or prediction made at the outset	6
No information about the setup	3
No measurement	7
Incomplete measurement	2
No controlled variable	1
No variable in the investigation	1
No variable manipulation	8
No reason provided (missing)	1
Total	29

The results above suggest that students held a very narrow understanding of what a scientific method is and often linked it to hypothesis testing, variable manipulation and measurement of several variables. For them, a non-manipulative parameter measurement scenario may not qualify as a scientific investigation.

## Conclusions and discussion

Summative assessments, especially in high-stakes contexts, determine what is valued for teaching and learning given that they define the parts of the curriculum that are awarded credit. Following recent educational reforms in England, summative assessments of practical science in GCSEs have been included in the written science papers. Hence, they became less reflective of the socio-constructivist theories that motivated their design in the early 1990s to give way to what is claimed to be more valid and reliable assessments (Ofqual 2013, 2015). While the new written assessment reforms addressed practical issues associated with previous formats of practical science assessments such as teacher workload and malpractice, the assessments can only be considered more valid if they reflected authentic scientific enquiry and can only be worthwhile if they encouraged deep learning of practical science that allowed students to demonstrate complex thinking. In this paper, we articulated how written assessments of practical science can be enhanced through the use of robust theoretical models that aimed to highlight the value and diversity of scientific methods. We presented a theoretical framework based on Brandon’s (1994) philosophical conceptualisation of the diversity of scientific methods and used this framework to design a series of practical science assessments. This empirical study illustrated how Year 10 students from two state schools in England engaged with and perceived the assessments.

The findings suggest that students found the assessment questions rather difficult. This finding was expected because of the younger age of the participants, the lower familiarity of the students with the type of questions and the relatively short teaching time of both, the physics concept (electrical circuits) and Brandon’s Matrix (i.e., the diversity of scientific methods). However, what was unexpected was students reporting that taking measurements and making conclusions were amongst their least favoured activities. Their reports of not enjoying taking measurements were incoherent with their reporting of enjoying setting up equipment and recording observations. This finding can be interpreted in terms of a distinction between the hands-on aspects of practical science (i.e. setting up equipment) which they report to enjoy versus minds-on aspects (i.e. taking measurements) which they do not particularly enjoy. This finding is consistent with those observed by Sharpe and Abrahams (2020) and furthermore, it provides further nuance about various aspects of the hands-on aspects of practical science in the context of an examination. These authors reported that secondary students’ attitudes to practical work were positive they were not constant and homogenous but change over time. The affective value of practical work was found to vary by subject although in all three sciences this value decreased as students approached their GCSE examinations. Many educational approaches advocate the use of hands-on practical activities in science lessons such as taking measurements (Satterthwait 2010). Students’ incoherent reports vis-à-vis hands-on practical activities are inconclusive and are difficult to interpret without additional data. Furthermore, there may be discrepancies between how students’ view practical science and the definition of practical science in terms of the emphasis on types of methods that were embedded in the assessments.

A surprising finding was related to how students felt about drawing conclusions from data. This is a particular skill that has been specified as important in documents such as the NGSS (NGSS Lead States 2013). It is a skill that has been promoted in the context of argumentation (i.e. justification of conclusions with evidence and reasons) in various school subjects (Erduran, Guilfoyle, and Park 2020). Hence, there are implications for not only science education but also cross-curricular reform to ensure consistency in how students are supported in drawing conclusions from observations and data.

The use of Brandon’s matrix allowed for framing curriculum standards and designing assessments in order to realise goals about practical science in general and scientific methods in particular. The results indicated that students held a very narrow understanding of what a scientific method is and often linked it to hypothesis testing, variable manipulation and measurement of several variables. For them, a non-manipulative parameter measurement scenario did not qualify as a scientific investigation. This outcome in itself illustrates the need for diversifying the scientific methods that are included in the design of summative assessments and ultimately in science teaching and learning. While science curriculum reforms continue in many parts of the world, it is imperative that high stakes assessments are aligned with curricular goals. For example, the South Korean science curriculum now lists ‘nature of scientific knowledge and method’ as a significant learning outcome (MOE, MSICT and KOFAC 2019, p. 19, 60) which coheres with the vision captured in the diversity of scientific methods presented in this paper. Although the study illustrates some shortcomings in terms of students’ reception and understanmuding of different methods in science, it is expected that through longer-term exposure in lessons as well as sustained professional development of their teachers, ultimately students’ perceptions, engagement and understanding will improve.

## References

[cit0001] Abrahams, I. 2009. “Does Practical Work Really Motivate? A Study of the Affective Value of Practical Work in Secondary School Science.” *International Journal of Science Education* 31 (17): 2335–2353. doi:10.1080/09500690802342836.

[cit0002] Abrahams, I. 2010. *Practical Work in Secondary Science: A Minds‐on Approach*. London: Continuum.

[cit0003] Abrahams, I., M. J. Reiss, and R. M. Sharpe. 2013. “The Assessment of Practical Work in School Science.” *Studies in Science Education* 49: 209–251. doi:10.1080/03057267.2013.858496.

[cit0004] Abrahams, I., and R. Millar. 2008. “Does Practical Work Really Work? A Study of the Effectiveness of Practical Work as A Teaching and Learning Method in School Science.” *International Journal of Science Education* 30: 1945–1969. doi:10.1080/09500690701749305.

[cit0005] Alderson, J. C., and D. Wall. 1993. “Does Washback Exist?” *Applied Linguistics* 14: 115–129. doi:10.1093/applin/14.2.115.

[cit0006] Au, W. 2007. “High-Stakes Testing and Curricular Control: A Qualitative Metasynthesis.” *Educational Researcher* 36: 258–267. doi:10.3102/0013189X07306523.

[cit0007] Baird, J.-A., D. Andrich, T. N. Hopfenbeck, and G. Stobart. 2017. “Assessment and Learning: Fields Apart?” *Assessment in Education: Principles, Policy and Practice* 24 (3): 317–350. doi:10.1080/0969594X.2017.1319337.

[cit0008] Bandura, A. 1969. *Principles of Behavior Modification*. New York, NY: Holt, Rinehart & Winston.

[cit0009] Berliner, D. 2011. “Rational Responses to High Stakes Testing: The Case of Curriculum Narrowing and the Harm that Follows.” *Cambridge Journal of Education: Mirrors for Research in Classrooms* 41 (3): 287–302. doi:10.1080/0305764X.2011.607151.

[cit0010] Black, P., and D. Wiliam. 1998a. “Assessment and Classroom Learning.” *Assessment in Education: Principles, Policy & Practice* 5: 7–74.

[cit0011] Black, P., and D. Wiliam. 1998b. *Inside the Black Box: Raising Standards through Classroom Assessment*. London: King’s College London School of Education.

[cit0012] Borghans, L., and T. Schils. 2019. “Decomposing Achievement Test Scores into Measures of Cognitive and Noncognitive Skills.” https://ssrn.com/abstract=3414156

[cit0013] Brandon, R. 1994. “Theory and Experiment in Evolutionary Biology.” *Synthese* 99: 59–73. doi:10.1007/BF01064530.

[cit0014] Childs, A., and J.-A. Baird. 2020. “General Certificate of Secondary Education (GCSE) and the Assessment of Science Practical Work: An Historical Review of Assessment Policy.” *The Curriculum Journal*. xx (xx): xxx–xxx. doi:10.1002/curj.20. PMC7566739 33100601

[cit0015] Clarkson, S. G., and D. K. Wright. 1992. “An Appraisal of Practical Work in Science Education.” *School Science Review* 74 (266): 39–42.

[cit0016] Coe, R. 2007. “Changes in Standards at GCSE and A‐level: Evidence from ALIS and YELLIS. Report for the Office of National Statistics,April 2007.”

[cit0017] Cullinane, A., S. Erduran, and S. J. Wooding. 2019. “Investigating the Diversity of Scientific Methods in High-Stakes Chemistry Examinations in England.” *International Journal of Science Education* 41 (16): 2201–2217. doi:10.1080/09500693.2019.1666216. 32165859 PMC7034329

[cit0018] Department for Education. 2015. “Biology, Chemistry and Physics GCSE Subject Content.” https://assets.publishing.service.gov.uk/government/uploads/system/uploads/attachment_data/file/593831/GCSE_single_science_formatted.pdf

[cit0019] Department for Education. 2017. “GCSE, AS and A Level Assessment Objectives.” https://www.gov.uk/government/publications/assessment-objectives-ancient-languages-geography-and-mfl/gcse-as-and-a-level-assessment-objectives#science

[cit0020] Donnelly, J. F., A. Buchan, E. W. Jenkins, P. Laws, and G. Welford. 1996. *Investigations by Order: Policy, Curriculum and Science Teachers’ Work under the Education Reform Act*. Driffield: Studies in Education.

[cit0021] El Masri, Y. H., S. Ferrara, P. W. Foltz, and J.-A. Baird. 2017. “Predicting Item Difficulty of Science National Curriculum Tests: The Case of Key Stage 2 Assessments.” *Curriculum Journal* 28 (1): 59–82. doi:10.1080/09585176.2016.1232201.

[cit0022] Ercikan, K. 2015. “This Issue.” *Theory into Practice* 54 (3): 179–182. doi:10.1080/00405841.2015.1044335.

[cit0023] Erduran, S. 2020. Reframing science education in light of the Covid-19 pandemic. *School Science Review* 102 (378): 38–43.

[cit0024] Erduran, S., and Z. R. Dagher. 2014. *Reconceptualizing the Nature of Science for Science Education : Scientific Knowledge, Practices and Other Family Categories*. Dordrecht, Netherlands: Springer.

[cit0025] Ferrara, S., and T. Duncan. 2011. “Comparing Science Achievement Constructs: Targeted and Achieved.” *The Educational Forum* 75 (2): 143–156. doi:10.1080/00131725.2011.552691.

[cit0026] Graesser, A. C., and D. S. McNamara. 2011. “Computational Analyses of Multilevel Discourse Comprehension.” *Topics in Cognitive Science* 3 (2): 371–398. doi:10.1111/j.1756-8765.2010.01081.x. 25164300

[cit0027] Graesser, A. C., D. S. McNamara, Z. Cai, M. Conley, H. Li, and J. Pennebaker. 2014. “Coh-Metrix Measures Text Characteristics at Multiple Levels of Language and Discourse.” *Elementary School Journal* 115: 210–229. doi:10.1086/678293.

[cit0028] Haladyna, T. M., and M. C. Rodriguez. 2013. *Developing and Validating Test Items*. New York, NY: Routledge.

[cit0029] Hofstein, A., and V. N. Lunetta. 2004. “The Laboratory in Science Education: Foundations for the Twenty-First Century.” *Science Education* 88 (1): 28–54. doi:10.1002/sce.10106.

[cit0030] House of Lords Science and Technology Committee. 2006. *Tenth Report of Session 2005-06 Science Teaching in Schools*.

[cit0031] James, M. 2008. “Assessment and Learning.” In *Unlocking Assessment. Understanding for Reflection and Application*, edited by S. Swaffield, 20–35. Abingdon: Routledge.

[cit0032] Jenkins, E. W. 1995. “Central Policy and Teacher Response? Scientific Investigation in the National Curriculum in England and Wales.” *International Journal of Science Education* 17 (4): 471–480. doi:10.1080/0950069950170406.

[cit0033] Kang, J., J. Hense, A. Scheersoi, and T. Keinonen. 2019. “Gender Study on the Relationships between Science Interest and Future Career Perspectives.” *International Journal of Science Education* 41 (1): 80–101. doi:10.1080/09500693.2018.1534021.

[cit0034] Krapp, A., and M. Prenzel. 2011. “Research on Interest in Science: Theories, Methods, and Findings.” *International Journal of Science Education* 33 (1): 27–50. doi:10.1080/09500693.2010.518645.

[cit0035] Kyriakides, L., and B. P. M. Creemers. 2009. “Teacher Behaviour and Student Outcomes: Suggestions for Research on Teacher Training and Professional Development.” *Teaching and Teacher Education* 25: 12–23. doi:10.1016/j.tate.2008.06.001.

[cit0036] Linn, R. L. 2000. “Assessments and Accountability.” *Educational Researcher* 29: 4–16.

[cit0037] Madaus, G. F., K. M. Russel, and J. Higgins. 2009. *The Paradoxes of High Stakes Testing: How They Affect Students, Their Parents, Teachers, Principals, Schools, and Society*. Charlotte, NC: Information Age Publishing .

[cit0038] McComas, W. F. 1998. “The Principal Elements of the Nature of Science: Dispelling the Myths.” In *The Nature of Science in Science Education*, edited by W. F. McComas, 53–70. Dordrecht: Kluwer Academic Publishers.

[cit0039] Millar, R., and J. F. Osborne. 1998. *Beyond 2000: Science Education for the Future*. London: King’s College London.

[cit0040] Ministry of Education (MOE), Ministry of Science and ICT (MSICT), & Korea Foundation for the Advancement of Science and Creativity (KOFAC). 2019. *Scientific Literacy for All Koreans: Korean Science Education Standards for the Next Generation*. Seoul: KOFAC.

[cit0041] Muijs, D., and D. Reynolds. 2010. *Effective Teaching. Evidence and Practice*. London: Sage.

[cit0042] National Research Council. 2012. *A Framework for K-12 Science Education: Practices, Crosscutting Concepts and Core Ideas*. Washington, DC: National Academies Press.

[cit0043] Neisser, U. 1967. *Cognitive Psychology*. New York, NY: Appleton Century Crofts.

[cit0044] NGSS Lead States. 2013. *Next Generation Science Standards: For States, by States*. Washington, DC: National Academies Press.

[cit0045] Ofqual. 2013. *Review of Controlled Assessment in GCSEs*. Coventry: Ofqual.

[cit0046] Ofqual. 2015. “Assessment of Practical Work in GCSE Science Analysis of Consultation Responses – March 2015.”

[cit0047] Osborne, J. 2015. “Practical Work in Science: Misunderstood and Badly Used?” *School Science Review* 96 (357): 16–24.

[cit0048] Pearson, P. D., A. M. Knight, M. A. Cannady, J. B. Henderson, and K. L. McNeill. 2015. “Assessment at the Intersection of Science and Literacy.” *Theory into Practice* 54 (3): 228–237. doi:10.1080/00405841.2015.1044372.

[cit0049] Pellegrino, J. W., M. R. Wilson, J. A. Koenig, and A. S. Beatty, eds. 2014. *Developing Assessments for the Next Generation Science Standards*. Washington, DC: National Academies Press. doi:10.17226/18409.

[cit0050] Popham, W. J. 1987. “The Merits of Measurement-driven Instruction.” *Phi Delta Kappan* 68: 679–682.

[cit0051] Reeve, C. L., E. D. Heggestad, and F. Lievens. 2009. “Modeling the Impact of Test Anxiety and Test Familiarity on the Criterion-Related Validity of Cognitive Ability Tests.” *Intelligence* 37 (1): 34–41. doi:10.1016/j.intell.2008.05.003.

[cit0052] Reiss, M., I. Abrahams, and R. Sharpe. 2012. *Improving the Assessment of Practical Work in School Science*. London: Gatsby Foundation.

[cit0053] Ryan, R. M., and E. L. Deci. 2000. “Intrinsic and Extrinsic Motivations: Classic Definitions and New Directions.” *Contemporary Educational Psychology* 25 (1): 54–67. doi:10.1006/ceps.1999.1020. 10620381

[cit0054] Ryoo, K., and M. C. Linn. 2015. “Designing and Validating Assessments of Complex Thinking in Science.” *Theory into Practice* 54 (3): 238–254. doi:10.1080/00405841.2015.1044374.

[cit0055] Sadler, P. M., G. Sonnert, Z. Hazari, and R. Tai. 2012. “Stability and Volatility of STEM Career Interest in High School: A Gender Study.” *Science Education* 93 (3): 411–427. doi:10.1002/sce.21007.

[cit0056] Satterthwait, D. 2010. “Why are ‘Hands-on’ Science Activities so Effective for Student Learning?” *Teaching Science* 56 (2): 7–10.

[cit0057] Schiefele, U., A. Krapp, and A. Winteler. 1992. “Interest as A Predictor of Academic Achievement: A Meta-Analysis of Research.” In *The Role of Interest in Learning and Development*, edited by K. A. Renninger, S. Hidi, A. Krapp, and K. A. Renn, 183–212. Hillsdale, NJ: Erlbaum.

[cit0058] Sharpe, R., and I. Abrahams. 2020. “Secondary School Students’ Attitudes to Practical Work in Biology, Chemistry and Physics in England.” *Research in Science & Technological Education* 38 (1): 84–104. doi:10.1080/02635143.2019.1597696.

[cit0059] Shepard, L. A. 2000. “The Role of Assessment in a Learning Culture.” *Educational Researcher* 29: 4–14. doi:10.3102/0013189X029007004.

[cit0060] Skinner, B. F. 1953. *Science and Human Behavior*. New York, NY: Macmillan.

[cit0061] Sternberg, R. J. 1981. “Testing and Cognitive Psychology.” *American Psychologist* 36: 1181–1189. doi:10.1037/0003-066X.36.10.1181.

[cit0062] Stobart, G., and T. Eggen. 2012. “High-Stakes Testing – Value, Fairness and Consequences.” *Assessment in Education: Principles, Policy & Practice* 19: 1–6.

[cit0063] Torrance, H., and J. Pryor. 1998. *Investigating Formative Assessment. Teaching, Learning and Assessment in the Classroom*. Buckingham: Open University Press.

[cit0064] Vygotsky, L. S. 1978. *Mind in Society*. Cambridge, MA: Harvard University Press.

[cit0065] Watson, J. B. 1930. *Behaviorism*. New York, NY: Norton.

[cit0066] Watson, J. R., J. R. L. Swain, and C. McRobbie. 2004. “Students’ Discussions in Practical Scientific Inquiries.” *International Journal of Science Education* 26 (1): 25–45. doi:10.1080/0950069032000072764.

[cit0067] Wiliam, D. 2010. “What Counts as Evidence of Educational Achievement? The Role of Constructs in the Pursuit of Equity in Assessment.” *Review of Research in Education* 34: 254–284. doi:10.3102/0091732X09351544.

[cit0068] Wivagg, D., and D. Allchin. 2002. “The Dogma of ‘The’ Scientific Method.” *The American Biology Teacher* 69 (9): 645–646.

[cit0069] Wooding, S., A. Cullinane, and S. Erduran. 2020. *Supporting the Teaching of Scientific Methods in Practical Science*. Oxford: University of Oxford. doi:10.5287/bodleian:xqvKxnmnX.

